# Whisker Velocity Patterns Tell Rats What They're Feeling

**DOI:** 10.1371/journal.pbio.0030036

**Published:** 2005-01-11

Whiskers don't fossilize, so it's hard to say when they first evolved. But it's quite likely they emerged along with mammals, over 200 million years ago. To elude the eye (and feet) of ungainly dinosaurs, it's thought these shrew-like prototypes foraged at night and sought refuge underground, where the sensory advantages of whiskers would come in handy. Nocturnal animals use whiskers much like the blind use walking sticks: to navigate their surroundings, explore close objects, and avoid running into things.

Whiskers, or vibrissae, connect to nerves, blood vessels, and muscles. These special connections allow rats, for example, to actively “whisk” the surface of objects and discern fine differences in texture, just as we move our fingertips along a surface to pick up details. In the wild, whisking helps rats navigate unfamiliar terrain to find food. But how does the brain know what the animal is touching?

Rat whiskers scan surfaces in a rhythmic motion that excites sensory receptor cells embedded in their whisker pad. Receptors in each whisker shaft are innervated by several hundred “first-order neurons” that relay sensory signals to second-order neurons in the brain stem, then on to third-order neurons in the thalamus, and finally on to the cortex, where sensory stimuli are integrated in cell clusters called barrels.

Ehsan Arabzadeh, Erik Zorzin, and Mathew Diamond work with rats to investigate how sensory receptors extract fundamental features from complex and diverse stimuli to encode texture. Not much is known about how receptor and cortical neurons respond to active whisking along irregular surfaces, though responses to simple stimuli (like sinusoidal vibrations) suggest that neurons might represent texture by encoding kinetic features of whisker vibrations, in particular, velocity. In a new study, Diamond and colleagues investigate the connection between textures, whisker vibrations, and neural codes: do distinct textures produce distinct vibrations? If so, how are these vibrations encoded and reported?[Fig pbio-0030036-g001]

**Figure pbio-0030036-g001:**
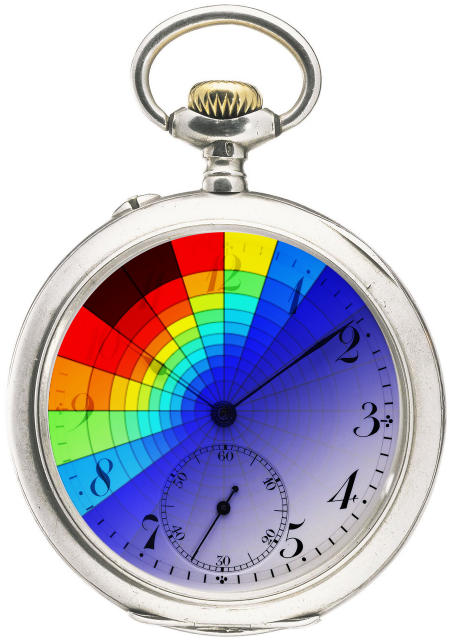
Timing of neuronal activity captures sensory information

The authors first collected kinetic data of whiskers moving across different textured surfaces. Stimulating cranial nerve VII of anesthetized rats (the motor nerve) generated whisking movements akin to those seen in conscious rats; the kinetics of these movements and the vibrations of the whisker shafts were measured under different conditions, including no contact with objects (“free whisk”), contact with smooth objects, and contact with various grades of sandpaper. These vibrations were then “played back” to other rats, while measuring the neuronal activity at two critical stages in the sensory pathway: the first-order neurons that innervate the whiskers and the barrel cortex neurons that integrate the incoming signal.

Altogether, the authors collected a neural dataset consisting of first-order recordings, barrel cortical cluster recordings, and simultaneous paired recordings from both sites, all in response to playback of the library of texture-related vibrations. This approach afforded the opportunity to directly compare encoding of information at both levels in the sensory pathway. These recordings show, the authors argue, that temporally distinct firing patterns in the trigeminal ganglion (the cell bodies of the first-order neurons) and cortex captured the kinetic features of the texture-induced vibrations. Each texture's “kinetic signature” is encoded by a characteristic, temporally precise firing pattern associated with whisker movement. Compared to free whisking, coarse sandpaper produced irregular bursts of high and low velocity, and both first-order and cortical neurons fired far more impulses for coarse sandpaper than for free whisks. The authors then used stimuli consisting of random velocities to uncover the “tuning curves” of neurons, and simulations showed that these neuronal tuning curves could perfectly predict the real neural responses to textures.

Noting the close match between the simulated and natural responses, Diamond and colleagues conclude that the texture-induced firing patterns observed in the first-order and cortical neurons suggest that neurons selectively encode elemental kinetic features—namely, high velocity—to tell rats what they're whisking. This selectivity allows even a single whisker to transmit significant bits of texture-specific information to the brain. Interesting as rat whisking may be, these findings have relevance beyond the world of whiskered beings, shedding light on the underlying neural processes that translate touch into recognition.

